# Hyperdense Pancreatic Ductal Adenocarcinoma: Clinical Characteristics and Proteomic Landscape

**DOI:** 10.3389/fonc.2021.640820

**Published:** 2021-02-25

**Authors:** He Xu, Jie Hua, Qingcai Meng, Xiaohong Wang, Jin Xu, Wei Wang, Bo Zhang, Jiang Liu, Chen Liang, Xianjun Yu, Si Shi

**Affiliations:** ^1^ Department of Pancreatic Surgery, Fudan University Shanghai Cancer Center, Shanghai, China; ^2^ Department of Oncology, Shanghai Medical College, Fudan University, Shanghai, China; ^3^ Shanghai Pancreatic Cancer Institute, Shanghai, China; ^4^ Pancreatic Cancer Institute, Fudan University, Shanghai, China; ^5^ Department of Radiology, Fudan University Shanghai Cancer Center, Shanghai, China

**Keywords:** pancreatic ductal adenocarcinoma, hyperdense, CT imaging, overall survival, protein landscape

## Abstract

**Purpose:**

Hypodensity of pancreatic ductal adenocarcinoma (PDAC) during contrast-enhanced computed tomography (CECT) examination is common, but a minority of PDAC patients exhibit hyperdense images. The present study examined the clinical characteristics and protein landscape of PDAC with hyperdensity.

**Materials and Methods:**

A total of 844 pathologically confirmed PDAC patients who underwent CECT before surgery were included. During the parenchymal phase of CECT, patients were assigned to the hyperdense or hypodense group based on CT values. Clinical and CT characteristics for predicting relapse-free survival (RFS) and overall survival (OS) were analyzed using the Kaplan–Meier method and Cox proportional hazards model. The expression of the tumor angiogenesis marker CD31 and stroma-related protein CTHRC1 were analyzed using immunohistochemistry (IHC) assay to evaluate differences between the two groups. Proteomics was performed to compare the possible mechanisms underlying the differential enhancement on CT scans.

**Results:**

Based on CECT, 43 and 801 PDAC patients had hyperdense and hypodense lesions, respectively. All 43 patients presented a hyperdense lesion in the parenchymal phase. The mean CECT values of the hyperdense group were higher than the hypodense group (102.5 ± 17.4 and 53.7 ± 18.7, respectively, *P* **<** 0.001). The hyperdense group had a better prognosis than the hypodense group (median RFS, 19.97 vs. 12.34 months, *P* = 0.0176; median OS, 33.6 vs. 20.3 months, *P* = 0.047). Multivariate analysis showed that age, higher CA19-9 levels (> 300 U/ml), tumor stage, tumor differentiation, tumor CT density, and adjuvant chemotherapy were significant independent prognostic factors for OS. CD31 immunohistochemical staining showed that the hyperdense PDACs had a higher microvessel density than the hypodense group (*P* **<** 0.001). CTHRC1 expression was higher in the hypodense group (*P* = 0.019). Sixty-eight differentially expressed proteins were found using the tandem mass tag labeling-based quantification of the proteomes of PDAC tissue samples, and 7 proteins (POFUT1, PKP2, P0DOX4, ITPR1, HBG2, IGLC3, SAA2) were related to angiogenesis.

**Conclusion:**

Patients who presented with a hyperdense mass on CECT had a higher microvessel density and better prognosis. Anti-angiogenic therapy may be suitable for these patients.

## Introduction

Pancreatic cancer is a lethal disease with a dismal prognosis, and its mortality rate is almost equal to its morbidity rate (1). The 5-year survival remains at 5%–8%, and it is expected to become the second leading cause of cancer-related death in 2030 (1, 2). Surgical resection is the only curative treatment, but less than 20% of patients are candidates for surgery at the time of diagnosis, and less than 20% of these patients survive 5 years after surgery (1). Therefore, early diagnosis and treatment are essential.

Computed tomography (CT), especially multidetector-row CT (MDCT) with a specific pancreatic protocol, is now performed routinely in most specialized hospitals for the diagnosis of pancreatic tumors. The National Comprehensive Cancer Network (NCCN) clinical practice guidelines in oncology have also recommended MDCT for the prediction of resectability and potential for reconstruction (3). Imaging examination plays a significant role in the treatment of patients with pancreatic adenocarcinoma (PDAC) (4). Hypo- or isoattenuation with main pancreatic duct dilation, abrupt cut-off of the pancreatic duct, and distal pancreatic atrophy can aid in the diagnosis of pancreatic cancer (5). The precision of the diagnosis and prediction of resectability is more than 85% (6).

Normal pancreatic tissue has a plentiful supply of blood, while cancerous pancreatic tissue has a decreased supply. Therefore, pancreatic adenocarcinoma often presents as a hypodense or isodense lesion compared to normal pancreatic tissue on contrast-enhanced CT (CECT) (7). However, we found that several patients with surgically confirmed PDAC presented hyperdense lesions on CT imaging. In addition, this group of patients had a better prognosis. This study aimed to investigate the differences between these two groups and try to identify the potential mechanism explaining the long survival time of patients with hyperdense pancreatic lesions.

## Materials and Methods

### Patients

**Figure 1:**
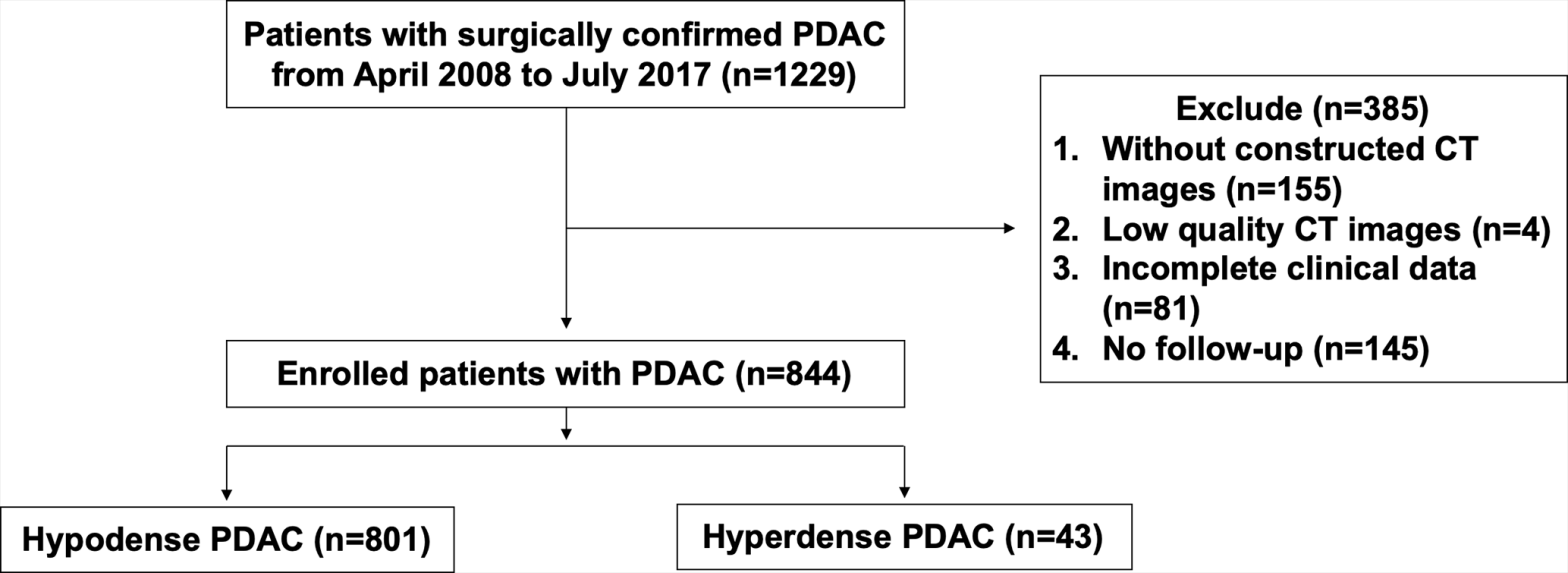
Flow diagram of patient enrollment.

A retrospective analysis was performed on PDAC patients with R0 resection from April 2008 to July 2017 in Fudan University Shanghai Cancer Center (FUSCC). None of the patients received neoadjuvant therapy before surgery. During this period, most of the patients received at least one pancreatic CT examination. PDAC patients were included if they met the following criteria: (a) patients who underwent CECT according to a specific pancreatic protocol within one week before surgery; (b) patients with high-quality imaging images (8); (c) patients with complete clinical data. We excluded 145 patients without follow-up among the 1229 eligible patients. Finally, 844 patients were enrolled. The flow diagram is presented in **Figure 1**. The Ethics Committee of FUSCC approved this retrospective study. Tissue samples were collected from patients who had provided written informed consent before the study.

### CT Technique

Patients were instructed to fast for more than 8 h prior to the CT scan. To better distinguish the gastrointestinal tract, most patients without contraindications drank 500 ml of water before the examination. Helical CT detections were performed at the radiology department of FUSCC (n=844) with MDCT scanners according to the pancreatic protocol. Three phase-contrast CT scans were performed at different time points. Iohexol was used at a high concentration as the intravenous contrast agent at an injection speed of 3 ml/s. The scan acquisition time windows were 30–35 s (arterial phase), 40–55 s (parenchymal phase), and 65–70 s (portal venous phase) after iohexol was administered. The axial section thickness ranged from 1 mm to 5 mm. Coronal, sagittal, and cross-sectional plane images were available for all patients.

### Image Analysis

CT images were reviewed independently by two pancreatic surgeons (HX and SS, > 5 years of pancreatic surgery experience) and one experienced abdominal radiologist (XW, > 10 years of experience reading abdominal CT images). The surgeons evaluated all the CT images as follows: When the difference between the maximum enhancement part of the tumors at the parenchymal phase and the surrounding normal pancreas was greater than 0 Hounsfield units (HU), the patients were assigned to the hyperdense group. Patients with other enhancement levels, including those with isodense tumors, were assigned to the hypodense group. The radiologist reviewed the images again to ensure that the patients in each group met the inclusion criteria. CT values for each lesion were measured three times, and the mean value was calculated. The final outcome was the mean of measurements of the 3 doctors. The tumor border and growth pattern data were not collected because this study mainly focused on the mass density in radiology.

### Clinical, Pathologic, and Follow-Up Data Collection

Features including age, sex, tumor location, tumor size, CA19-9 level, adjuvant chemotherapy, tumor stage, carcinoma embolus, perineural invasion, and CT values were collected from the patient medical records at FUSCC. Tumor-node-metastasis (TNM) stage was assessed according to the American Joint Committee on Cancer (AJCC), 8^th^ edition. All enrolled patients were diagnosed with PDAC based on routine postoperative pathological reports. The patients were regularly followed-up by telephone or face-to-face interviews. Surgeons and radiologists were blinded to the patient information before data collection.

### Immunohistochemistry (IHC)

Paraffin wax specimens were selected from 78 patients (hyperdense group vs. hypodense group, 39 vs. 39) for IHC staining. There was no statistically significant difference in the baseline data between the two groups. All tumor samples were obtained during radical surgical resection. Each specimen was fixed in 10% formalin for 24 h before paraffin embedding. Each tissue sample was stained with hematoxylin-eosin (HE) to assess morphological changes. The IHC protocol was as follows: (a) deparaffinization of xylene and rehydration of gradient ethanol; (b) microwave-mediated thermal repair for 10 min in pH 6.0 citric acid (BL604A, Biosharp, China); (c) 3% H_2_O_2_-mediated blocking of endogenous peroxidase at 37°C for 20 min; (d) incubation with a CD31 antibody (11265-1-AP, 1:1,000, Proteintech, China) or a CTHRC1 antibody (16534-1-AP, 1:50, Proteintech, China) at 4°C overnight; (e) incubation with anti-rat/rabbit immunohistochemical detection antibody (GTVisionTM III Detection System/Mo&Rb, GK500710, Gene Tech, China) at 37°C for 1 h; (f) DAB coloration and hematoxylin counterstaining; and (g) dehydration in gradient ethanol and xylene.

Digital microphotographs (200×) were taken using a microscope (Olympus, Japan). The area of positive cells was scored as 0 (<10%), 1 (10 ≤ positive cells < 25%), 2 (25% ≤ positive cells < 50%), 3 (50% ≤ positive cells < 75%), or 4 (≥ 75%). The intensity was scored as 0 (negative), 1 (low), 2 (moderate), or 3 (strong). The product of these two scores represented the final expression level. Scores ≥ 6 were considered high expression, and scores <6 indicated low expression. The final scores were the mean of two experts in pathology.

### Proteomics Sequencing and Bioinformatics Methods

A total of 10 tissue samples (hyperdense vs. hypodense, 5 vs. 5) were used for proteomics sequencing. The samples were ground in liquid nitrogen into cell powder and digested with trypsin. After digestion, the peptides were desalted on a Strata X C18 SPE column (Phenomenex) and vacuum dried. Peptides were reconstituted and processed according to tandem mass tag (TMT) labeling. The labeled peptides were analyzed by liquid chromatography-mass spectrometry (LC-MS), and the resulting LC-MS data were processed using the MaxQuant search engine. For Gene ontology (GO) enrichment analysis, proteins were classified into three categories *via* GO annotation: biological process, cellular compartment and molecular function. For each category, a double-tailed Fisher’s exact test was designed to test the enrichment of the differentially expressed protein against that of all identified proteins. GO terms with a corrected *P* value < 0.05 were considered statistically significant.

### Statistical Analysis

All statistical analyses were performed with SPSS (version 25.0, IBM, NY, USA). The values are presented as the mean ± standard deviation. Differences between groups were calculated by Pearson χ^2^ test, Fisher’s exact test or Mann-Whitney *U* test. Kaplan-Meier curves and log-rank tests were analyzed for survival comparison. Univariate or multivariate analyses for overall survival (OS) were evaluated using a Cox proportional hazards model. Differences were considered statistically significant at a two-sided *P* < 0.05.

## Results

### Clinicopathological Features

**Figure 2:**
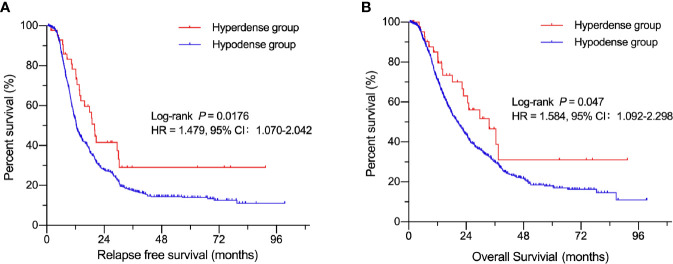
Kaplan-Meier curves of patients in the hyperdense and hypodense groups. **(A)** Comparison of RFS between the hypo- and hyperdense groups. **(B)** Comparison of OS between the hypo- and hyperdense groups.

A total of 844 patients (498 males, 59.0%; 346 females, 41.0%) with surgically and pathologically confirmed PDACs were enrolled in this study. The median age of onset was 61 years old (range, 29–84). Forty-three patients presented with high-density lesions when compared to normal pancreas tissues on parenchymal phase CT images. These 43 patients were regarded as the hyperdense group. The mean tumor size in this group was 3.3 ± 1.4 cm (range, 1.0–11.2 cm). No statistically significant differences were found in terms of age, sex, adjuvant chemotherapy, CA19-9 level, tumor stage, or carcinoma embolus between the hyperdense and hypodense groups (**Table 1**). However, the hyperdense group had a higher percentage of carcinomas at the head of the pancreas (*P* < 0.001) and a higher perineural invasion percentage (*P* = 0.036) than the hypodense group. Patients in the hyperdense group had a better RFS and OS than those in the hypodense group (median RFS, 19.97 vs. 12.34 months, *P* = 0.0176; median OS, 33.6 vs. 20.3 months, respectively, *P* = 0.047, **Figure 2**).

**Table 1:** Patients and tumor characteristics.

Variables	*N*	Hypodense	Hyperdense	*P* value
Total (%)	844	801 (94.9)	43 (5.1)	
OS (months)	21.0	20.3	33.6	0.047
CT values (parenchymal phase)				
PDAC	–	53.7 ± 18.7	102.5 ± 17.4	<0.001
Normal pancreas	–	89.6 ± 36.6	83.2 ± 14.1	0.250
Age (years), n (%)				0.987
<65	529	502 (62.7)	27 (61.4)	
≥65	315	299 (37.3)	16 (38.6)	
Gender, n (%)				0.164
Male	498	477 (59.6)	21 (48.8)	
Female	346	324 (40.4)	22 (51.2)	
Location, n (%)				<0.001
Head	495	456 (56.9)	39 (90.7)	
Body and tail	349	345 (43.1)	4 (9.3)	
Adjuvant chemotherapy,n (%)				0.359
Yes	659	623 (77.8)	36 (83.7)	
No	185	178 (22.2)	7 (16.3)	
Tumor stage, n (%)				0.736
I	353	336 (41.9)	17 (39.5)	
II	403	383 (47.8)	20 (46.5)	
III	88	82 (10.2)	6 (14.0)	
CA19-9, n (%)				0.829
<300 U/ml	504	479 (59.8)	25 (58.1)	
≥300 U/ml	340	322 (40.2)	18 (41.9)	
Carcinoma embolus				0.135
Yes	213	198 (24.7)	15 (34.9)	
No	631	603 (75.3)	28 (65.1)	
Perineural invasion				0.036
Yes	737	695 (86.8)	42 (97.7)	
No	107	106 (13.2)	1 (2.3)	

### Prognostic Factors for Resected Pancreatic Cancer

**Table 2** shows the relationship between clinicopathologic features and RFS. **Table 3** shows the relationship between clinicopathologic features and OS. Univariate analysis revealed that RFS was significantly related to tumor stage, tumor differentiation, adjuvant chemotherapy, perineural invasion, carcinoma embolus and CA 19-9 level, and OS was significantly related to tumor stage, tumor differentiation, adjuvant chemotherapy, perineural invasion, and CA 19-9 level. The hyperdense group patients tended to have a better RFS (*P* = 0.0176) and OS (*P* = 0.047). The multivariate analyses showed that the following features are independent prognostic factors for OS: age > 65 years (hazard ratio [HR] = 1.209, *P* = 0.040), no adjuvant chemotherapy (HR = 1.481, *P* < 0.001), CA19-9 level > 300 U/ml (HR = 1.509, *P* < 0.001), tumor stage (HR = 1.415, *P* < 0.001, stage II; HR = 2.512, *P* < 0.001, stage III), and tumor differentiation (HR = 1.902, *P* = 0.011, moderate; HR = 2.656, *P* < 0.001, poor). Additionally, density classification was an independent prognostic factor (HR = 1.772, *P* = 0.017). RFS and OS had the same trends in multivariate analyses.

**Table 2:** Univariate and multivariate analyses for the RFS.

Variables	*n*	Univariate analysis			Multivariate analyses		
		HR	95% CI	*P* value	HR	95% CI	*P* value
**Age (years)**							
**< 65**	529	1			1		
**≥ 65**	315	1.119	0.951–1.316	0.174	1.153	0.977–1.360	0.093
**Gender**							
**Male**	498	1			1		
**Female**	346	0.880	0.749–1.033	0.117	0.901	0.766–1.061	0.211
**Location**							
**Head**	495	1			1		
**Body and tail**	349	0.903	0.770–1.059	0.211	0.942	0.799–1.111	0.479
**Adjuvant chemotherapy**							
**Yes**	659	1			1		
**No**	185	1.353	1.118–1.637	0.002	1.321	1.087–1.605	0.005
**Tumor stage**							
**I**	353	1			1		
**II**	403	1.446	1.221–1.712	<0.001	1.350	1.135–1.605	0.001
**III**	88	2.277	1.750–2.963	<0.001	2.104	1.600–2.766	<0.001
**CA 19-9**							
**< 300 U/ml**	504	1			1		
**≥ 300 U/ml**	340	1.576	1.343–1.849	<0.001	1.473	1.250–1.736	<0.001
**Carcinoma embolus**							
**Yes**	213	1			1		
**No**	631	0.760	0.636–0.908	0.003	0.869	0.719–1.049	0.144
**Perineural invasion**							
**Yes**	737	1			1		
**No**	107	0.745	0.586–0.948	0.017	0.764	0.599–0.974	0.030
**Tumor differentiation**							
**Well**	40	1			1		
**Moderate**	517	1.965	1.278–3.023	0.002	1.879	1.217–2.901	0.004
**Poor**	287	2.421	1.561–3.757	<0.001	2.331	1.497–3.627	<0.001
**Enhancement**							
**Hyperdense**	43	1			1		
**Hypodense**	801	1.616	1.083–2.411	0.019	0.550	0.364–0.828	0.004

**Table 3:** Univariate and multivariate analyses for the OS.

Variables	*n*	Univariate analysis			Multivariate analyses		
		HR	95% CI	*P* value	HR	95% CI	*P* value
**Age (years)**							
**< 65**	529	1			1		
**≥ 65**	315	1.189	0.996–1.419	0.056	1.209	1.009–1.450	0.040
**Gender**							
**Male**	498	1			1		
**Female**	346	0.854	0.716–1.019	0.080	0.884	0.739–1.057	0.176
**Location**							
**Head**	495	1			1		
**Body and tail**	349	0.911	0.765–1.086	0.300	0.947	0.791–1.135	0.557
**Adjuvant chemotherapy**							
**Yes**	659	1			1		
**No**	185	1.502	1.222–1.845	<0.001	1.481	1.199–1.828	<0.001
**Tumor stage**							
**I**	353	1			1		
**II**	403	1.497	1.241–1.805	<0.001	1.415	1.169–1.712	<0.001
**III**	88	2.613	1.978–3.450	<0.001	2.512	1.880–3.356	<0.001
**CA 19-9**							
**< 300 U/ml**	504	1			1		
**≥ 300 U/ml**	340	1.607	1.349–1.914	<0.001	1.509	1.261–1.807	<0.001
**Carcinoma embolus**							
**Yes**	213	1			1		
**No**	631	0.848	0.694–1.037	0.108	0.992	0.802–1.225	0.937
**Perineural invasion**							
**Yes**	737	1			1		
**No**	107	0.759	0.585–0.983	0.037	0.770	0.593–1.000	0.050
**Tumor differentiation**							
**Well**	40	1			1		
**Moderate**	517	2.020	1.239–3.295	0.005	1.902	1.162–3.114	0.011
**Poor**	287	2.721	1.656–4.472	<0.001	2.656	1.610–4.383	<0.001
**Enhancement**							
**Hyperdense**	43	1			1		
**Hypodense**	801	1.586	1.003–2.508	0.049	1.772	1.105–2.840	0.017

### Imaging and IHC Features

**Figure 3:**
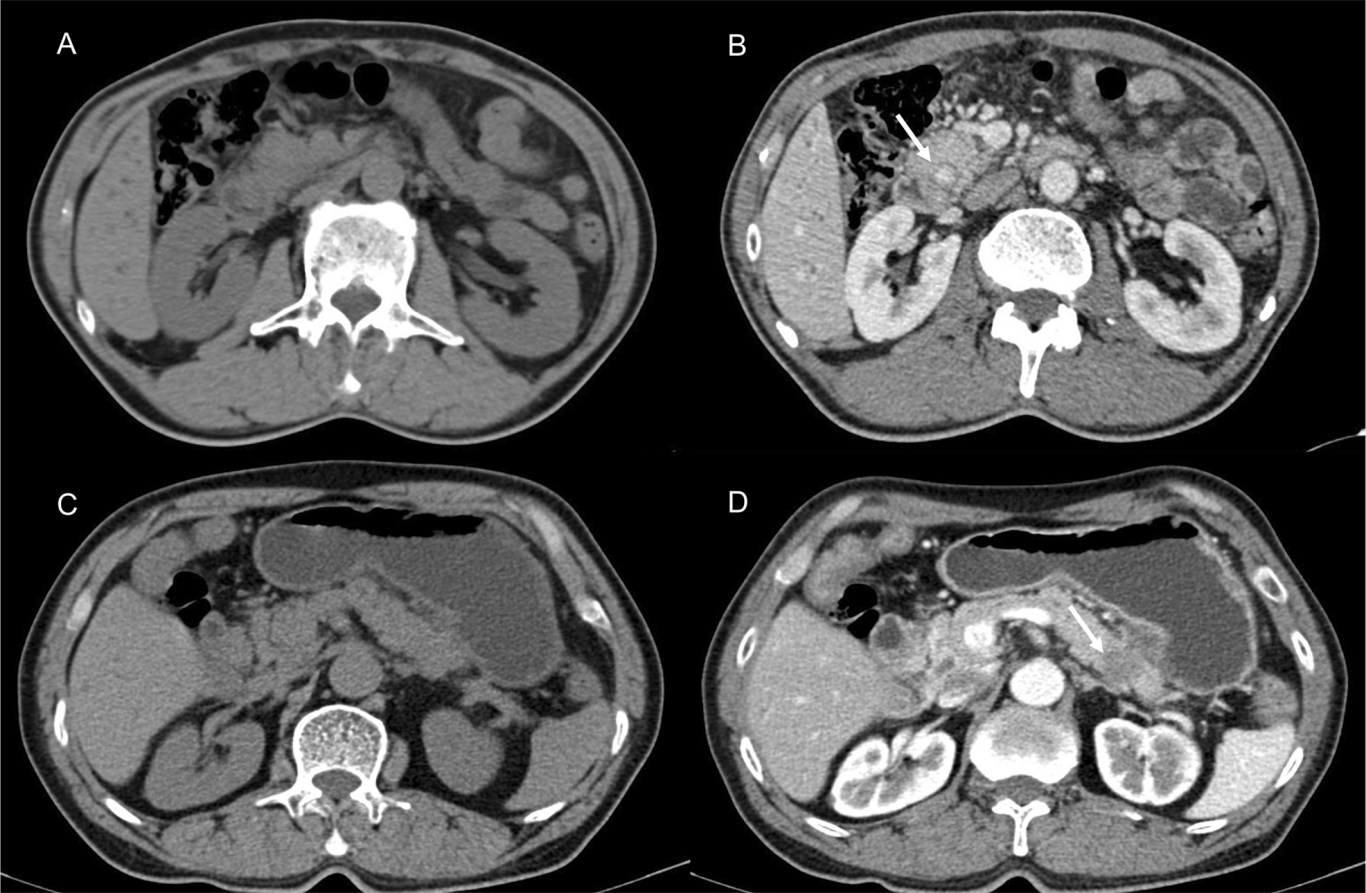
Presentive CECT images of PDAC. **(A, B)** CECT revealed a lesion in the head of the pancreas. The tumor [**(B)**, white arrow] showed higher density in the parenchymal phase. **(C, D)** Typically, hypodense PDAC tumors present in the tail of the pancreas [**(D)**, white arrow].

**Figure 4:**
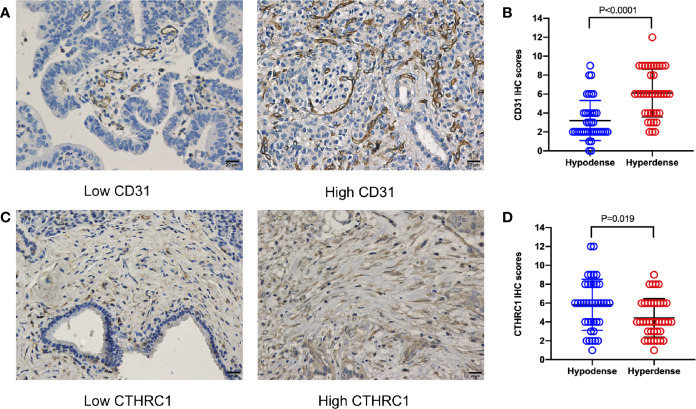
CD31 and CTHRC1 IHC staining in PDAC. **(A)** Representative images of PDAC samples according to CD31 expression (scale bar: 20 μm). **(B)** Statistical results of the CD31 expression comparison between the hypo- and hyperdense groups using the Mann-Whitney U test. **(C)** Representative images of PDAC samples according to CTHRC1 expression (scale bar: 20 μm). **(D)** Statistical results of the CTHRC1 expression comparison between the hypo- and hyperdense groups using the Mann-Whitney U test.

Sample CT images of hyperdense and hypodense PDAC are shown in **Figures 3A–D**. The mean CT value in the parenchymal phase of these pancreatic cancers was 56.2 HU (3 HU - 140 HU, range). The tumors of patients in the hyperdense group had a higher mean CT value than those in the hypodense group (102.5 ± 17.4 HU vs. 53.7 ± 18.7 HU, respectively, *P* < 0.001). Of all the hyperdense group patients, 39 patients had paraffin wax specimens. We randomly matched these patients to 39 patients in the hypodense group. There were no significant differences in age, sex or tumor stage between the two groups. HE and CD31 staining were also performed on the serial sections. Twenty-six patients (67%, 26/39) in the hyperdense group exhibited high expression. However, only 7 patients (18%, 7/39) in the hypodense group exhibited high expression. The difference in CD31 expression between the two groups was statistically significant (*P* < 0.001, **Figures 4A, B**).

### TMT Outcomes of Patient Characteristics

**Figure 5:**
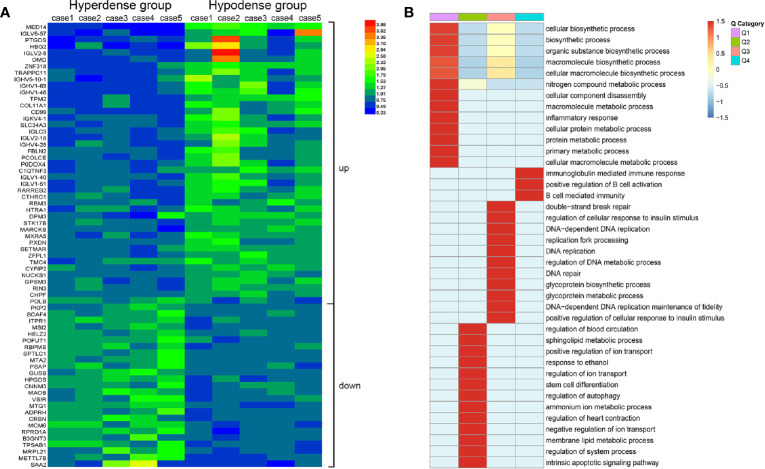
Total protein TMT labeling was used to quantify the proteomes of pancreatic cancer tissue samples. **(A)** Differential protein expression between hyperdense patients and hypodense patients. **(B)** Heat map of cluster analysis based on GO classification.

According to the proteomics results, 318,938 secondary spectrograms were obtained by mass spectrometry. After the theoretical protein data were searched, the number of available effective spectrograms was 59,113, and the utilization rate of spectrograms was 18.5%. A total of 5,548 proteins were detected (**Supplementary Table 1**).When the *P* value was < 0.05, a change in differential expression over 1.3-fold was used as the threshold for significant upregulation, and a fold change of less than 1/1.3 was used as the threshold for significant downregulation. In total, 68 differentially expressed proteins (42 upregulated vs. 26 downregulated; **Figure 5** and **Supplementary Table 2**) were found. GO classification, Kyoto Encyclopedia of Genes and Genomes (KEGG) pathway, and protein domain enrichment analyses were carried out for the differentially expressed proteins in each comparison group, with the aim of determining whether the differentially expressed proteins showed significant enrichment of specific functions. Based on the enrichment analysis results, the *P* values from Fisher’s exact test and the identified enriched functions of the different groups were assessed by the hierarchical clustering method and illustrated as a heatmap. Finally, we found that seven differentially expressed proteins (POFUT1, PKP2, P0DOX4, ITPR1, HBG2, IGLC3, and SAA2) were related to angiogenesis, seven differentially expressed proteins (MAOB, MARCKS, PKP2, RBM3, COL11A1, ITPR1, POLB) were related to perineural invasion, and three differentially expressed proteins (PCOLCE, COL11A1 and CTHRC1) were associated with stroma (**Supplementary Table 2**). CTHRC1 was selected to verify the results of TMT sequencing. The expression of CTHRC1 was significantly higher in the hypodense group (*P* = 0.019, **Figures 4C, D**), which was the same as the TMT sequencing.

## Discussion

With limited progress in the treatment of PDAC, the most promising strategy for improving patient survival is early detection and effective drug therapy. CT is the most popular noninvasive examination for the primary diagnosis and treatment of PDAC (9) and has played a central role in the decision-making process for PDAC treatment (10). Compared to magnetic resonance imaging (MRI), CT scans are more popular due to their low cost and convenience. Typical PDAC often presents as a hypodense or isodense lesion in the pancreas. Some indirect signs, such as main pancreatic duct dilation, abrupt cut-off of the pancreatic duct, distal pancreatic atrophy, and enlarged lymph nodes, aid in pancreatic cancer diagnosis (6, 11). However, a small group of PDACs present as hyperdense lesions, in contrast with the typical observation. Our data have revealed that patients with hyperdense lesions may have a better OS than those with hypo- or isodense lesions. Therefore, we anticipate a greater focus on identifying these individuals, determining the differences between these patients and those with hypodense lesions, exploring the potential mechanisms of lesion hyperdensity and, ultimately, seeking individualized treatment strategies.

In this study, we retrospectively collected all the CT reports that were made available as a part of routine radiological work at our hospital. The CT review showed a high concordance rate (100%) in CT density between pancreatic surgeons and radiologists, enabling us to confirm the quality of the research. A total of 5.1% of patients (43/844 in parenchymal phases) presented with pancreatic lesions that had a higher density than that of the normal pancreas, and these patients were selected as the hyperdense group. Compared to the hypodense group, the 43 PDACs in the hyperdense group had smaller diameters (hyperdense group vs. hypodense group; 2.9 ± 1.1 cm vs. 3.3 ± 1.5 cm; *P* = 0.041). This may be one reason why the hyperdense group had a better prognostic trend.

CT density plays a significant role in the diagnosis and prognostic prediction of PDAC (12, 13). However, to the best of our knowledge, this research is the first to discuss the features of hyperdense PDACs. According to the multivariate analyses, our study demonstrated that patients in the hyperdense group had a better prognosis than those in the hypo- or isodense groups. The hyperdense PDACs from these 43 patients were variable in terms of tumor stage. We found that 39.5% (17 of 43) were stage I, 46.5% (20 of 43) were stage II, and 14.0% (6 of 43) were stage III. On the basis of the enhancement of PDACs, our outcomes are in concordance with previous research on PDACs, which showed that 75% (15 of 20) of PDACs exhibit hypodensity on dynamic CT (4). Age, tumor differentiation and stage, adjuvant chemotherapy, and CA19-9 levels were also identified as independent prognostic risk factors. CA19-9 is currently the best tumor marker for diagnosing patients, monitoring therapy, determining resectability and assessing the prognosis of pancreatic cancer (14, 15). Further mechanistic studies on CA19-9 assessment combined with CT imaging evaluation may improve the early diagnosis of pancreatic tumors.

The density of lesions manifested on contrast CT is the main feature used for the diagnosis of pancreatic tumors. Different density patterns may point to different diseases. Usually, PDAC presents as a hypo- or isodense lesion, while pancreatic neuroendocrine tumor (pNET) presents as a hyperdense lesion. However, previous studies have proven that pancreatic tumors do not manifest in the typical manner for many reasons (8, 16). At this time, improving the methods used for differential diagnosis is very important for pancreatic surgeons. This study urges surgeons and radiologists to pay more attention to hypervascular pancreatic lesions to avoid misdiagnosis.

In 1971, Folkman et al. proposed the hypothesis that tumor growth depends on angiogenesis (17). This claim was subsequently confirmed and is now considered to be one of the major features of cancer (18). In general, the more blood supply one tissue has, the higher CT values it presents. Our CD31 IHC staining results confirmed this statement. CD31 is a well-known vascular endothelial marker. Studies have shown that high expression of CD31 in tumors, such as pNETs, is related to a worse prognosis (19). However, the results of our study led to the opposite conclusion. Patients with high CD31 expression had a better OS than those with low CD31 expression. Typically, hypovascular tumors with a relatively high microvessel density are associated with a better prognosis. This is consistent with previous bioinformatics reports (20). Tumor stroma is a key component of pancreatic cancer. An increase in stroma reduces the efficacy of chemotherapy drugs (21). The present study showed that the expression of CTHRC1, a stroma related protein, was higher in the hypodense group. Further studies are needed to verify whether the stroma affects CT density in PDAC.

For a deeper understanding of the different manifestations of the two groups of patients, we performed proteomic analysis. In total, 68 differentially expressed proteins were found, seven of which were related to blood vessel formation. This may account for the difference in vessel density between the two groups. High vascularity is known to be related to malignancy in many tumors (19, 22, 23). Increased expression of CD31 is significantly associated with increased tumor size and lymph-vascular invasion in Merkel cell carcinoma (22). Anti-angiogenic therapy is an established treatment approach for many solid tumors. However, in this study, patients with relatively high vascular density had a better prognosis than those with low vascular density. This may correlate with changes in the tumor microenvironment, such as inevitably diminished drug delivery caused by a reduction in tumor blood vessels. The differentially expressed proteins POFUT1 (24) and PKP2 (25) correlate with angiogenesis and fibrosis in other cancer species. Anti-angiogenic therapy may be more effective for hyperdense PDAC patients than for hypodense PDAC patients. Furthermore, we may examine how these proteins and genes affect angiogenesis in PDAC in the future. Promoting vascular normalization in hypodense PDAC patients is a promising therapeutic strategy that could complement traditional antiangiogenic therapies.

There are several limitations to this study. First, the hyperdense group was small, and further large-scale studies are required to confirm the outcomes. Second, the mechanism behind the prognostic value of hyperdensity remains unclear. Further studies and additional clinical research are needed.

In conclusion, we demonstrated that the hypervascular presentation on CT was positively associated with OS. Anti-angiogenic therapy may be suitable for these patients. This novel finding would be helpful in the diagnosis of pancreatic cancer. Additional studies might be beneficial for supporting our conclusions and for illuminating the underlying mechanisms.

## Data Availability Statement

The datasets presented in this study can be found in online repositories. The names of the repository/repositories and accession number(s) can be found in the article/**Supplementary Material**.

## Ethics Statement

The studies involving human participants were reviewed and approved by Ethics Committee of Fudan University Shanghai Cancer Center. The patients/participants provided their written informed consent to participate in this study.

## Author Contributions

SS and XY designed the study. HX, JH, and QM contributed to the data collection and writing of the manuscript. HX, SS, and XW reviewed the CT imaging. JX, WW, BZ, JL, and CL provided technical support. SS and XY proofread the final version. All authors contributed to the article and approved the submitted version.

## Funding

This study was funded by the National Natural Science Foundation of China (nos. 81802352, 81772555, and 81902428), the National Science Foundation for Distinguished Young Scholars of China (No. 81625016), the Shanghai Sailing Program (nos. 19YF1409400 and 20YF1409000), the Shanghai Rising-Star Program (No. 20QA1402100), the Shanghai Anticancer Association Young Eagle Program (No. SACA-CY19A06), the Clinical and Scientific Innovation Project of Shanghai Hospital Development Center (nos. SHDC12018109 and SHDC12019109), and the Scientific Innovation Project of Shanghai Education Committee (No. 2019-01-07-00-07-E00057).

## Conflict of Interest

The authors declare that the research was conducted in the absence of any commercial or financial relationships that could be construed as a potential conflict of interest.
